# *EZH2* G553C significantly increases the risk of brain metastasis from lung cancer due to salt bridge instability

**DOI:** 10.1186/s12935-024-03362-w

**Published:** 2024-05-19

**Authors:** Hanjun Wang, Ling Wang, Sheng Zhang, Qicai Liu, Feng Gao

**Affiliations:** 1 Department of Pathology, The First Affiliated Hospital, Fujian Medical University, Fuzhou, 350004 People’s Republic of China; 2 Department of Pathology, Women’s Hospital, Zhejiang University School of Medicine, Hangzhou, China; 3 Department of Reproductive Medicine Centre, 1st Affiliated Hospital, Fujian Medical University, 20 Chazhong Road, Fuzhou, 350005 China

**Keywords:** *EZH2*, Lung cancer brain metastasis, NGS, Gene polymorphism, Forecast

## Abstract

**Background:**

The incidence and mortality of lung cancer is the highest in China and the world. Brain is the most common distant metastasis site of lung cancer. Its transfer mechanism and predictive biomarkers are still unclear. *EZH2* participates in the catalysis of transcriptional inhibition complex, mediates chromatin compactness, leads to the silencing of its downstream target genes, participates in the silencing of multiple tumor suppressor genes, and is related to cell proliferation, apoptosis and cycle regulation. In physiology, *EZH2* has high activity in stem cells or progenitor cells, inhibits genes related to cell cycle arrest and promotes self-renewal. To detect the expression and mutation of *EZH2* gene in patients with brain metastasis of lung cancer, and provide further theoretical basis for exploring the pathogenesis of brain metastasis of lung cancer and finding reliable biomarkers to predict brain metastasis of lung cancer.

**Methods:**

This study investigated susceptible genes for brain metastasis of lung cancer. The second-generation sequencing technology was applied to screen the differential genes of paired samples (brain metastasis tissues, lung cancer tissues and adjacent tissues) of lung cancer patients with brain metastasi.

**Results:**

It revealed that there was a significant difference in the G553C genotype of *EZH2* between lung cancer brain metastasis tissues and lung cancer tissues (*p* = 0.045). The risk of lung cancer brain metastasis in G allele carriers was 2.124 times higher than that in C allele carriers. Immunohistochemistry showed that compared with lung cancer patients and lung cancer patients with brain metastasis, the expression level of *EZH2* in lung cancer tissues of lung cancer patients was significantly higher than that in adjacent lung tissues (*p* < 0.0001), and higher than that in brain metastasis tissues (*p* = 0.0309). RNA in situ immunohybridization showed that *EZH2* mRNA expression was gradually high in lung cancer adjacent tissues, lung cancer tissues and lung cancer brain metastasis tissues.

**Conclusions:**

*EZH2* G553C polymorphism contributes to the prediction of brain metastasis of lung cancer, in which G allele carriers are more prone to brain metastasis.

**Supplementary Information:**

The online version contains supplementary material available at 10.1186/s12935-024-03362-w.

## Background

Lung cancer is one of the most common malignant tumors, and its morbidity and mortality remain high worldwide. Globally, lung cancer is the second most common cancer and the most common cause of cancer death. In 2020, a total of 1.8 million new death cases were attributable to lung cancer, accounting for 18% of all cancer mortality [1].

Lung cancer has high invasiveness and distant metastasis, which is one of the important reasons for poor treatment effect and high mortality of patients [2]. According to the cohort study, the incidence of brain metastasis is high when lung cancer is distant metastasis. Five years after the diagnosis of primary lung cancer, the incidence of brain metastasis is as high as 16.3%, and the prognosis of patients with brain metastasis is poor, with a median survival of only 3.1 to 12 months [3]. Treatment options are extremely limited, so preventing the occurrence of brain metastasis is of great significance to improve the outcome of lung cancer patients.

At present, the mechanism of brain metastasis includes metastasis cascade and tumor stem cell hypothesis [4], seed soil theory [5], gene driven theory [6], brain microenvironment influence, etc. [7], but the specific mechanism is still unclear, and the difficulty of establishing lung cancer brain metastasis model makes it difficult to carry out brain metastasis research, and many studies are still in the exploratory stage.The blood–brain barrier plays an important role in the process of brain metastasis. The migration of cancer cells across the endothelial barrier is a key step in brain metastasis. Studies have found that *EZH2*, as a major enzyme catalyzing H3K27me3, can affect angiogenesis by controlling gene expression in endothelial cells, and endothelial *EZH2* can destroy the integrity of blood–brain barrier. Therefore, the role of *EZH2* mutation in blood–brain barrier may provide a new breakthrough for brain metastasis.

Zeste homolog 2 enhancer (*EZH2*) is a key component of polycomb inhibitory complex 2 (PCR2), which has histone methyltransferase activity. The complex methylates lysine 27 (H3K27) of histone H3 to promote transcriptional silencing [8]. *EZH2* is mutated repeatedly in a variety of cancers and plays an important role in tumor proliferation and progression [9, 10]. *EZH2* is closely related to the occurrence and poor prognosis of a variety of tumors. Evidence shows that mutation or overexpression of *EZH2* is an important factor leading to tumor growth and migration. However, the current research basically discusses the relationship between it and primary cancer, and rarely involves metastasis or brain metastasis of lung cancer. In physiology, *EZH2* has high activity in stem cells or progenitor cells. *EZH2* inhibits genes related to cell cycle arrest and promotes self-renewal. *EZH2* plays an important role in epigenetic gene silencing, which usually depends on its histone methyltransferase activity. A large number of studies have shown that *EZH2*, as the catalytic subunit of PRC2 complex, catalyzes H3K27me3. Then PRC combines with H3K27me3 and lysine 119 of histone H2 (H_2AK119-UB1) to form a ternary complex. As a transcription inhibitor, the complex mediates chromatin compactness, resulting in the silencing of its downstream target genes. *EZH2* typically silences multiple tumor suppressor genes through its histone lysine methyltransferase activity. The target genes involved in cell proliferation, apoptosis and cell cycle regulation include INK4B-ARF-INK4A, Bim, TRAIL, KLF2, MSMB, FOXC1, hDAB2IP, etc. In addition, *EZH2* has an activation point mutation on Y641, A677 or A687 residues, resulting in increased methyltransferase activity and increased H3K27me3 level.

We screened metastatic cancer, carcinoma in situ and their corresponding adjacent tissues by second-generation sequencing and detected the presence of high-frequency mutations in *EZH2* in lung cancer primary lesions and brain metastatic tissues. Then we verified *EZH2* genotype by Sanger sequencing and found that *EZH2* G553C polymorphism may be associated with metastasis. Then the expression of *EZH2* protein and mRNA was detected by immunohistochemistry and RNA in situ hybridization, and the relationship between *EZH2* gene polymorphism, mRNA and protein expression and clinicopathological parameters of lung cancer brain metastasis was discussed, and the relationship between *EZH2* G553C polymorphism and lung cancer brain metastasis was studied.

## Methods and materials

### Tissue samples and clinical data collection

Paraffin tissue from brain metastases of lung cancer from the tumor tissue bank of the First Affiliated Hospital of Fujian Medical University from January 2019 to February 2022 was included in the study.Inclusion criteria: 1. patients who underwent lung surgery; 2. have complete clinical data and follow-up data; 3. sign the informed consent. Patients with any comorbidities or previous malignancies were excluded from the study.The relevant information of lung cancer patients with brain metastasis was collected, including age, gender, TNM stage, surgical method, prognosis, chemotherapy regimen, pathological grade, imaging characteristics and tumor marker level.A total of 48 male patients and 30 female patients ranging between 29–80 (median: 59) years were included in the study. Of these, 17 (21.7%) were smokers, four (5.13%) had SCLC, 74 (94.87%) had lung adenocarcinoma.Besides, 71 (91.03%) patients had single primary lung cancer and 7 ( 8.97%) patients had multiple primary lung cancer.See Table 1 for details of patients.This study was approved by the Ethics and Research Committee of the First Affiliated Hospital of Fujian Medical University and complies with the Helsinki Declaration.

**Table 1:** Distribution of *G553C* genotype of *EZH2* gene in brain metastases and lung cancer tissues

	Number of cases	Genotype			HWE	X^2^	P
		GG(%)	CG(%)	CC(%)			
Lung cancer brain metastasis tissue	78	3(3.8)	26(33.3)	49(62.82)	0.038		
Lung cancer tissue	60	2(3.3)	9(15)	49(81.7)	2.999		
	138	5	35	98		6.2150	0.045

### High throughput sequencing

The samples submitted for examination are matched lung cancer, paracancerous, and brain metastatic tissues from the Cancer Tissue Library of the First Affiliated Hospital of Fujian Medical University from 2019 to 2021. The samples include 5 pairs of brain metastatic tissues, lung cancer tissues, and lung cancer paracancerous tissues (> 5 cm away from the tumor tissue), all of which are lung adenocarcinoma brain metastases.High-throughput sequencing the second generation sequencing of tumor related mutant genes (Aiquanwai V2 Tumor Kit, product number: AIEC) was carried out by Aijitaikang Biotechnology Co., Ltd. (Beijing, China).

### Genotyping

DNA extraction was performed using a tumor paraffin tissue DNA extraction kit (Tiangen Biotechnology Co., Ltd.), and multiple sequencing and analysis were performed by Shenggong Biotechnology Co., Ltd.Design and synthesis of primers DSNP was selected from the NCBI genebank database to find the sequence of *EZH2*rs2302427 gene. The software Primer 5.0 was used to design forward and reverse primers. The primer blast in NCBI was used to evaluate and compare the designed primers, and its specificity was tested. The primer was located in *EZH2* (NG_032043.1): The software Primer 5.0 was used to design forward and reverse primers. The primer blast in NCBI was used to evaluate and compare the designed primers, and its specificity was tested. The primer was located in *EZH2* (ng_032043.1): The forward primer of PCR is 5'-GTTCGCACACCATTCAAA-3', and the reverse primer is 5 '-GACATTTGATGGCGTTAGAATG -3'. PCR condition is 95 ℃ undergoes initial denaturation for 10 min, followed by 35 cycles at 95 ℃for 30 s, 55 ℃ for 39 s, 72 ℃ for 1 min, and finally extended for 10 min at 72 ℃. The PCR product was then run on 1% agarose gel in 1X TAE buffer and purified to the target gene. Gene sequencing was performed using the ABI PRISM7700 sequencer (PerkinElmer, Inc.).After sequence analysis, the results were found in the result group, and the sequence analysis software was used for genotype comparison.

### Immunohistochemistry and HE staining

Hematoxylin and eosin (H&E) staining and immunohistochemistry (IHC) were performed on the brain metastatic tumor tissue of lung cancer.All tissues were taken into 8-micron sections, and *EZH2* polyclonal antibody (NOVUS company, product number: Lot HN0824, dilution is 1:6000) and FAM92B polyclonal antibody (NOVUS company, product number: Lot R38194, dilution is 1:5000) were immunolabeled on slides. The antigen was repaired using high-temperature pretreatment, and phosphate buffer saline was used as the negative control instead of the first antibody. Obtain IHC staining results for all tissues using the Motic digital slice scanning system. Under the same background brightness conditions, randomly select 5 fields of view for each tissue at high magnification (400x). Analyze the staining results using Image Pro Plus software. After importing the image and correcting the optical density, select the area to be measured, measure the cumulative optical density (IOD) of this area, and calculate the area of the measurement area (Area). The protein expression level is equivalent to the average IOD/Area of optical density. Take the average optical density values obtained from the measurements of five fields of view.

### RNA in situ hybridization

The *EZH2* RNA In Situ Hybridization Fluorescence Detection Kit (product number: GF003-50T) was purchased from Servicebio Company, and the testing steps were carried out according to the Servicebio Company RNA In Situ Hybridization Fluorescence Detection Kit User Manual. mRNA in situ heteropositivity is located in the nucleus or cytoplasm, with a staining pattern of green fluorescent particles.Tissue sections of brain metastases from lung cancer with different genotypes were selected. Enter xylene I 15 min-xylene II 15 min-absolute ethanol I 5 min-absolute ethanol II 5 min -85% ethanol 5 min -75% ethanol 5 min-nuclease free water in turn to dehydrate the tissue, immerse the tissue slices in 1 × repair solution, place the antigen repair box in a water bath with the temperature increased to 100°C in advance, continue to keep the temperature until 1 × repair solution boils and maintains for 15 min, then take out the antigen repair box, cool it naturally to room temperature, and clean it for 3 times with 1 × PBS for 5 min each time. The tissues were circled with immunohistochemical strokes, and the tissues were covered with proteinase K solution. The tissues were digested at 37 ℃ for 20–30 min, and then the slices were washed three times with 1 × PBS for 5 min each time. Add 40 °C pre heated hybridization solution to cover the sample, and place the wet box in the pre heated hybridization instrument to incubate at 40 °C for 30 min. Pour out the hybridization solution, drip about 60μL of the target probe mixture 1 hybridization solution preheated at 40 °C (the probe design is shown in the table) to cover the sample, and then place the wet box in the hybridization instrument at 40 °C for incubation for 3 h. The hybridization solution of target probe mixture 1 was poured out, and the slices were rinsed at 40 °C with 2 × SSC, 1 × SSC, 0.5 × SSC and 0.1 × SSC preheated at 40 ℃ for 5 min each. Drop about 60μL of target probe mixture 2 hybridization solution preheated at 40 °C to cover the sample, and put the wet box in the hybridization instrument at 40 °C to incubate for 45 min. The target probe mixture 2 hybridizing solution was poured out, and the slices were rinsed at 40 °C for 5 min at 2 × SSC, 1 × SSC, 0.5 × SSC and 0.1 × SSC preheated at 40 ℃. Add the fluorescent signal probe hybridization solution (IF488 probe (200 ×))preheated at 37 °C dropwise for about 60 μL cover the sample, and incubate the wet box in the hybridizer at 37 °C for 45 min. The slices were rinsed at 37 ℃ for 5 min with 2 × SSC, 1 × SSC, 0.5 × SSC and 0.1 × SSC preheated at 37 °C. After 1 × PBS moistening and washing for one time, and slightly drying, add 200-500 μL DAPI working solution to the circle to completely cover the tissue, and stain at room temperature without light for 8 min. After slightly drying DAPI dye solution, add anti fluorescence quenching agent dropwise for sealing. Observe the slices with a standard fluorescence microscope, and detect the fluorescence signal through the corresponding fluorescence detection channel under an appropriate multiple (20 × − 40 ×) objective lens.

**Table:** 

Name	Target sequence(5'-3')
EZH2-1	TGAGCTGTCTCAGTCGCATGTACTCTGA
EZH2-2	GATTTCCGTTCTTTCCAAAATTTTCTGACG
EZH2-3	GATGTGCACAGGCTGTATCCTTCGCTG
EZH2-4	ATGACTTGTGTTGGAAAATCCAAGTCACTG
EZH2-5	TACATTATGGGTACTGAAGCAACTGCATTC
EZH2-6	CATCTTCCACCATAAAATTCTGCTGTAGGG
EZH2-7	ACATTCTCTATCCCCGTGTACTTTCCCATC
EZH2-8	GGCATTCACCAACTCCACAAAAATTTCATC
EZH2-9	CGTCTCCATCATCATCATCGTCATCATCA
EZH2-10	TCTCGGTGATCCTCCAGATCTTTCTGCT
EZH2-11	TTTATCAGAAGGAAATTTCCGAGGTGGGC
EZH2-12	TCTGCTGTGCCCTTATCTGGAAACATTGA
EZH2-13	CTGGGAGCTGCTGTTCGGTGAGTTC
EZH2-14	TTTAGCATTTGGTCCATCTATGTTGGGGGT
EZH2-15	ACATCGCCTACAGAAAAGCGTATGAAAGG
EZH2-16	ATAAGTGTTGGGTGTTGCATGAAAAGGATG
EZH2-17	CCAAATGCTGGTAACACTGTGGTCCACA
EZH2-18	GGGTCTTTATCCGCTCAGCGGTGAGA
EZH2-19	TGCTGGGCCTGCTACTGTTATTGGGAA
EZH2-20	CCCTGCTTCCCTATCACTGTCTGTATCCT

### Population genetics analysis

ENSEMBL database (https://asia.ensembl.org/index.html), ICGC database (https://dcc.icgc.org/) search for data on genotype and gene frequency of *EZH2* high-frequency mutation sites in different countries and regions in the normal population.The gene frequencies of *EZH2* rs2302427C/G in different races and regions of the world were searched in ENSEMBL (ENSG000006462) database. There are five regions in East Asia, including Europe, America, Africa, South Asia and China. Then in the East Asia database, it subdivides China into three regions: Beijing Han people, Xishuangbanna Dai people and Southern Han people.

### Amino acid sequence conservation analysis

Six species close to humans were selected, including Brain metastatic(Human), Chlorocebus aethiops,Gorilla,Ncmarcus leucogenys,Pan paniscus, and Pan troglodytes in NCBI database (https://www.ncbi.nlm.nih.gov/).The amino acid sequences were compared by Mega7.0 software, and the comparison map was constructed by GeneDoc.

### Construction of protein spatial structure

Uniprot database (https://www.uniprot.org) Search for the structure of the protein PRC2 encoded by *EZH2* and construct the protein structure using PyMOL 2.5.4 to predict the impact of rs2302427 site on the encoded protein.

### Statistical analysis

Statistical analysis of the detection results was conducted using SPSS (version 17.0) and Graph Pad Prism (version 9.0) software.The correlation between *EZH2* genotype and clinical pathological parameters of patients was analyzed using t-test and chi square test; Pearson and Fisher tests were used to compare the frequency distribution of different genotypes in the lung cancer group and the lung cancer brain metastasis group; Kaplan Meier analysis of the relationship between gene polymorphism and survival in lung cancer patients with brain metastasis; The relative risk is represented by odds ratio (OR) and 95% CI (confidence interval).

## Results

### High-throughput sequencing found that *EZH2* mutation was closely related to brain metastasis of lung cancer

**Fig. 1:**
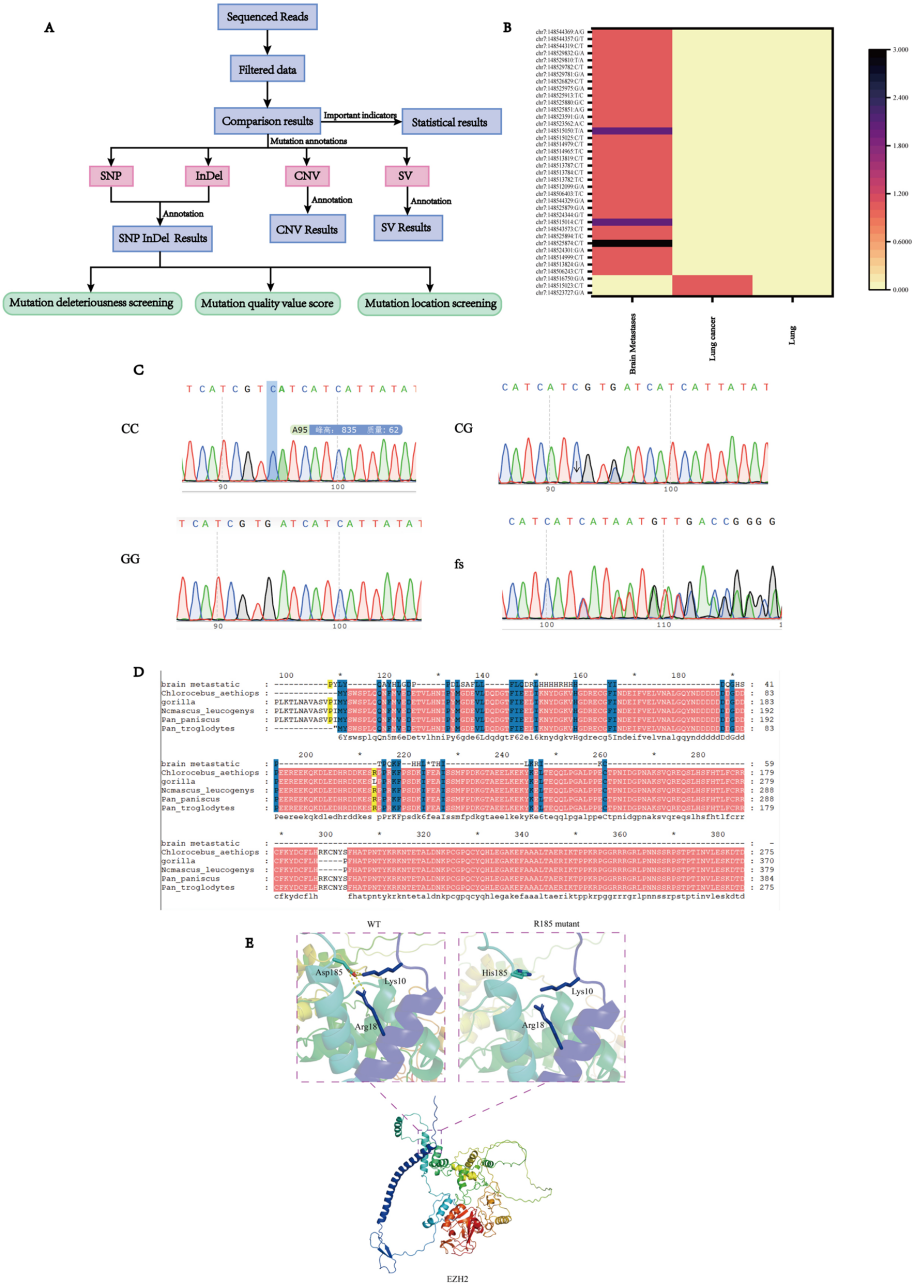
*EZH2* mutation found by high-throughput sequencing is closely related to brain metastasis of lung cancer. **A** High throughput screening flowchart, **B** Heat map of second-generation sequencing *EZH2* mutation site results, **C** Sanger sequencing diagram, **D** Species conservation, **E** Structure prediction

NGS technology was used to sequence brain metastasis tissue samples, lung cancer tissue samples, and lung cancer adjacent tissue samples from 5 patients with lung cancer brain metastasis (Fig. 1), and screen differential genes (Fig. 1A). It was found that 14 genes with multiple mutations (n ≥ 3) were unique to metastatic tumors, including DYDC1, KRTAP5-4, RECQL, FAM92B, ABCA8, PRAMEF12, NBPF10, IGFN1, BUB1, SMC6, STAT4, *EZH2*, RP1L1, and ATRX. *EZH2* was the most frequent with 38 mutations. FAM92B involved in endocytosis, vesicular fission and fusion, and actin cytoskeleton regulator, as well as the gene *EZH2* that plays a role in hematopoietic and nervous systems were identified. After comparing the NGS sequencing results of each group of samples, only 3 sites of *EZH2* mutation were found in the primary lesion of lung cancer, while 20 mutation sites were found in metastatic cancer tissue. Among them, G553C (Asp185His) was the site with the highest mutation frequency after brain metastasis (Fig. 1B). Moreover, FAM92B showed negative expression in brain metastases, primary lesions, and adjacent tissues, while *EZH2* showed positive expression in the same tissues (Supplemental Fig. 1).

### Verify the genotype frequency of rs2302427 C/G (*EZH2* G553C)

In order to verify the results of second-generation sequencing, DNA was extracted from 78 lung cancer brain metastatic tissues and 60 lung cancer non metastatic patients, and Sanger sequencing and *EZH2* were performed in parallel_ G553C genotype analysis showed that out of 78 cases of lung cancer with brain metastasis, 49 cases were CC genotype, 26 cases were CG genotype, and 3 cases were GG genotype; Among the 60 tissues of non-metastatic lung cancer patients as controls, 49 were CC genotype, 9 were CG genotype, and 2 were GG genotype (Fig. 1C).

### Analysis of amino acid sequence conservation and protein structure domain at G553C site of *EZH2*

For multiple alignment of *EZH2* protein sequences from six species, Asp185 is conservative (Fig. 1D). D185 forms a salt bridge with R18 and K10 before the G553C site mutation of *EZH2* gene, but H185 destroys all interactions after the mutation, presumably leading to a decrease in local stability (Fig. 1E).

### Functional prediction of G553C locus in *EZH2*

*EZH2* G553C, also known as the corresponding rs2302427 variant, is located in the 185th amino acid of *EZH2*, where the amino acid changes from Asp to His. PolyPhen-2 (http://genetics.bwh.harvard.edu/pph2/). Based on physical and comparative considerations, it is predicted that this variant has a 60.3% chance of affecting *EZH2*. This variant is also predicted to have functionality, with an AA change score of 85 (score range 0–215) in MutationTaster2.

### Hardy weinberg equilibrium analysis of genotype G553C

X^2^ test showed that the genotype frequency distribution of G553C conformed to Hardy Weinberg balance (Hardy Weinberg balance test in lung cancer brain metastasis group: X^2^ = 0.038, *p* = 0.9810; Hardy Weinberg balance test in lung cancer group: X^2^ = 2.9993, *p* = 0.2232). There was a significant difference between *EZH2* G553C genotype lung cancer brain metastasis and lung cancer population (*p* = 0.045) (Table 1), that is, there was a significant difference in G allele frequency between lung cancer brain metastasis and lung cancer (*p* = 0.031), The risk of lung cancer brain metastasis increased by 2.1241 times in G allele carriers OR = 2.124, 95% CI [1.061-4.254] (Table 2).

**Table 2:** Distribution of *EZH2* gene G553C gene frequency in lung cancer brain metastasis tissues and lung cancer tissues

	N	Allele frequency		OR [95%CI]	X^2^	P
		G (%)	C (%)			
Lung cancer brain metastasis tissue	156	32 (20.51)	124 (79.49)			
Lung cancer tissue	120	13 (10.8)	107 (89.2)			
	276	45	231	2.124 [1.061 ~ 4.254]	4.6569	0.031

### Population genetics analysis of G553C locus of *EZH2*

**Fig. 2:**
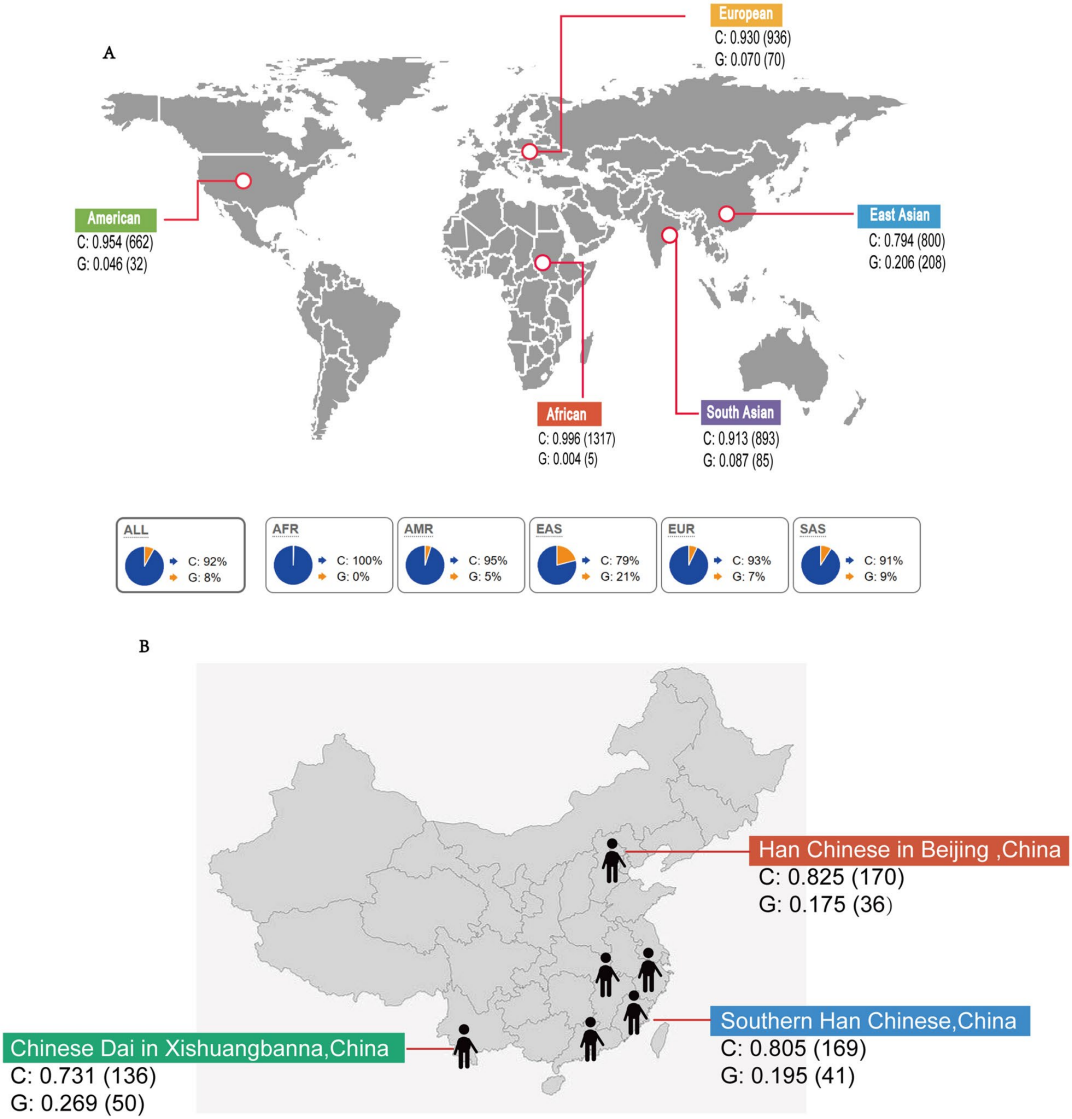
Population genetics analysis of *G553C* locus of *EZH2*. **A** Distribution Map of *EZH2_G553C* Gene Frequency in Five Global Regions, **B** Distribution Map of *EZH2_G553C* Gene Frequency in China

Search the global gene frequency of *EZH2* rs2302427C/G in the ENSEMBL database. Among them, there are 936 cases of allele C in the European region with a gene frequency of 0.930, and 70 cases of allele G with a gene frequency of 0.070; there are 662 cases of allele C in the United States, with a gene frequency of 0.954; there are 32 cases of allele G, with a gene frequency of 0.046; there are 1317 cases of allele C in Africa, with a gene frequency of 0.996; there are 5 cases of allele G, with a gene frequency of 0.004; In South Asia, there are 893 allele C cases with a gene frequency of 0.913, and 85 allele G cases with a gene frequency of 0.087; there are 800 cases of allele C in East Asia, with a gene frequency of 0.794; there are 208 cases of allele G, with a gene frequency of 0.206; among the global population, there are 4608 cases of allele C with a gene frequency of 0.920, 400 cases of allele G with a gene frequency of 0.080. Among them, East Asia, where China is located, has the highest G allele frequency among the five regions (Fig. 2A).

In the East Asian database, the distribution of the G553 gene frequency of the *EZH2* gene in three regions of China is included. There are 170 cases of allele C in the Han population of Beijing, with a gene frequency of 0.825; there are 36 cases of allele G, with a gene frequency of 0.175; there are 136 Dai people in Xishuangbanna with allele C and gene frequency of 0.731, and 50 with allele G and gene frequency of 0.269; there are 169 cases of allele C in the southern Han population, with a gene frequency of 0.825; there are 41 cases of allele G, with a gene frequency of 0.195 (Fig. 2B).

### The relationship between G553C polymorphism of *EZH2* and clinical pathological characteristics

Perform chi square test analysis on 78 lung cancer patients with brain metastasis of different genotypes, and record all chi square values and *p* values. There were no statistically significant differences (*p* > 0.05) in the gender composition, age, smoking history, pathological type, and whether there were multiple lung and brain lesions among the study subjects (Table 3).

**Table 3:** Correlation between *G553C* polymorphism of *EZH2* gene and clinical pathological characteristics of brain metastasis in lung cancer

C553G	Number of cases	Genotype			X^2^	P	Allele frequency		X^2^	P
		CC%	CG%	GG%			C%	G%		
Gender					2.194	0.334			1.204	0.272
Male	48	33 (68.750)	13 (27.083)	2 (4.167)			79 (82.292)	17 (17.708)		
Female	30	16 (53.333)	13 (43.333)	1 (3.333)			45 (75)	15 (25)		
Age (years)					0.457	0.796			0.347	0.556
< 60	40	24 (60.000)	14 (35.000)	2 (5.000)			62 (77.500)	18 (22.500)		
≥ 60	38	25 (65.789)	12 (31.579)	1 (2.631)			62 (81.333)	14 (18.667)		
Smoking history					2.484	0.289			0.899	0.343
No	61	36 (59.016)	23 (37.705)	2 (3.278)			95 (77.869)	27 (22.131)		
Yes	17	13 (76.471)	3 (17.647)	1 (5.882)			29 (85.294)	5 (14.706)		
Pathological type					0.283	0.868			0.002	0.967
Lung adenocarcinoma	73	46 (63.014)	24 (32.876)	3 (4.109)			116 (79.452)	30 (20.548)		
Small cell carcinoma of the lung	5	3 (60.000)	2 (40.000)	0 (0.000)			8 (80.000)	2 (20.000)		
Is there multiple lung diseases					1.688	0.430			0.509	0.475
No data	36	23 (63.889)	12 (33.333)	1 (2.778)			58 (80.556)	14 (19.444)		
Single shot	35	22 (62.857)	12 (34.286)	1 (2.857)			56 (80.000)	14 (20.000)		
Pilosity	7	4 (57.143)	2 (28.571)	1 (14.286)			10 (71.429)	4 (28.571)		
Is there multiple occurrences in the brain					1.895	0.388			1.611	0.204
Single shot	58	39 (67.241)	17 (29.310)	2 (3.448)			95 (81.897)	21 (18.103)		
Pilosity	20	10 (50.000)	9 (45.000)	1 (5.000)			29 (72.500)	11 (27.500)		

### *G553C* polymorphism of *EZH2* and its relationship with immunohistochemistry

**Fig. 3:**
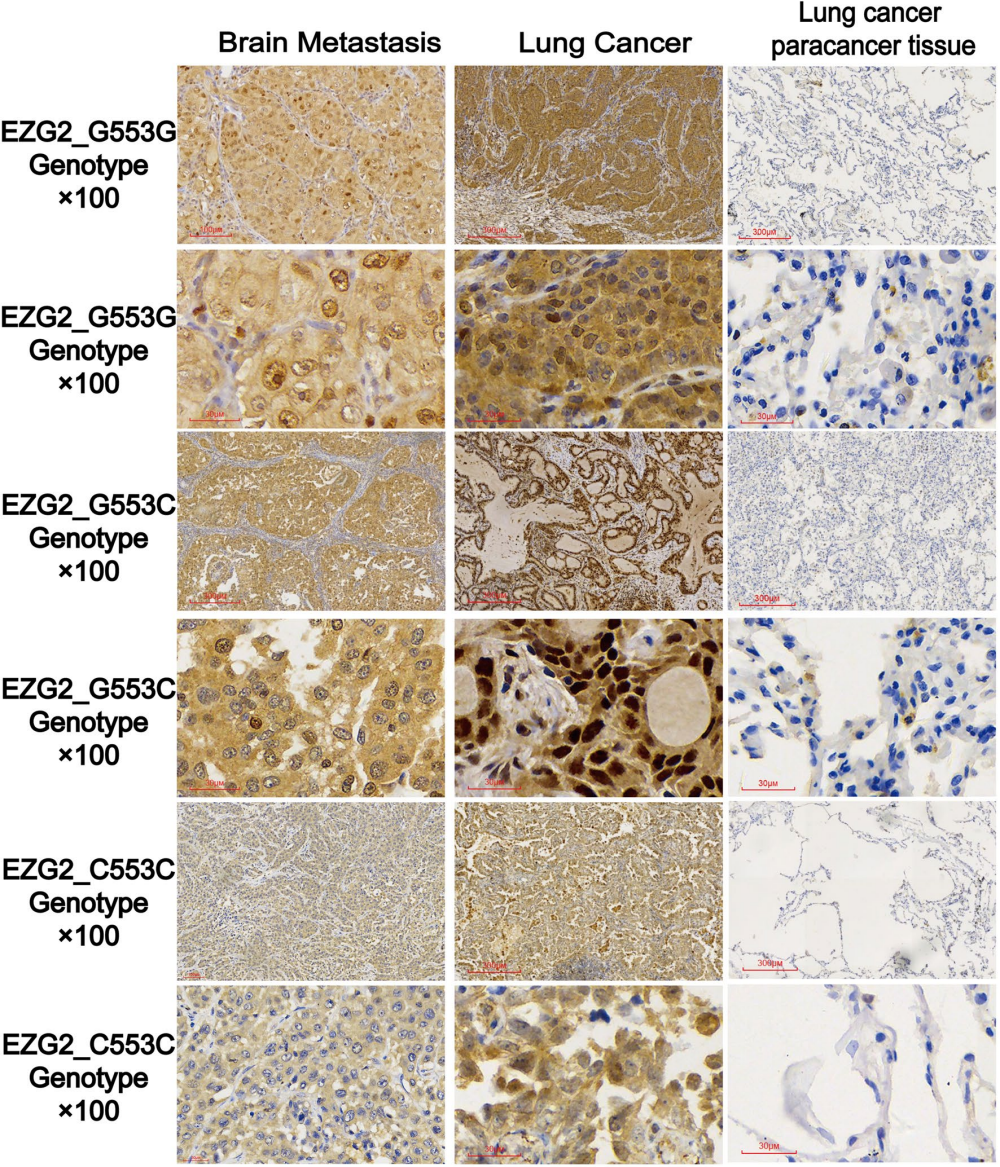
*EZH2 G553C* genotype and *EZH2* immunohistochemistry results in brain metastases, lung cancer, and normal lung tissue

Immunohistochemical detection of *EZH2* expression was performed on brain metastatic tissues, primary lung cancer lesions, and adjacent tissues of different genotypes of lung cancer. *EZH2* is negative in tissues adjacent to lung cancer. In lung cancer tissues and lung cancer brain metastases, *EZH2* is mainly expressed in the cytoplasm and nucleus, showing diffuse strong positivity, and there is no significant difference in expression between genotypes (Fig. 3).

### The relationship between G553C polymorphism of *EZH2* and survival time in lung cancer patients with brain metastasis

**Fig. 4:**
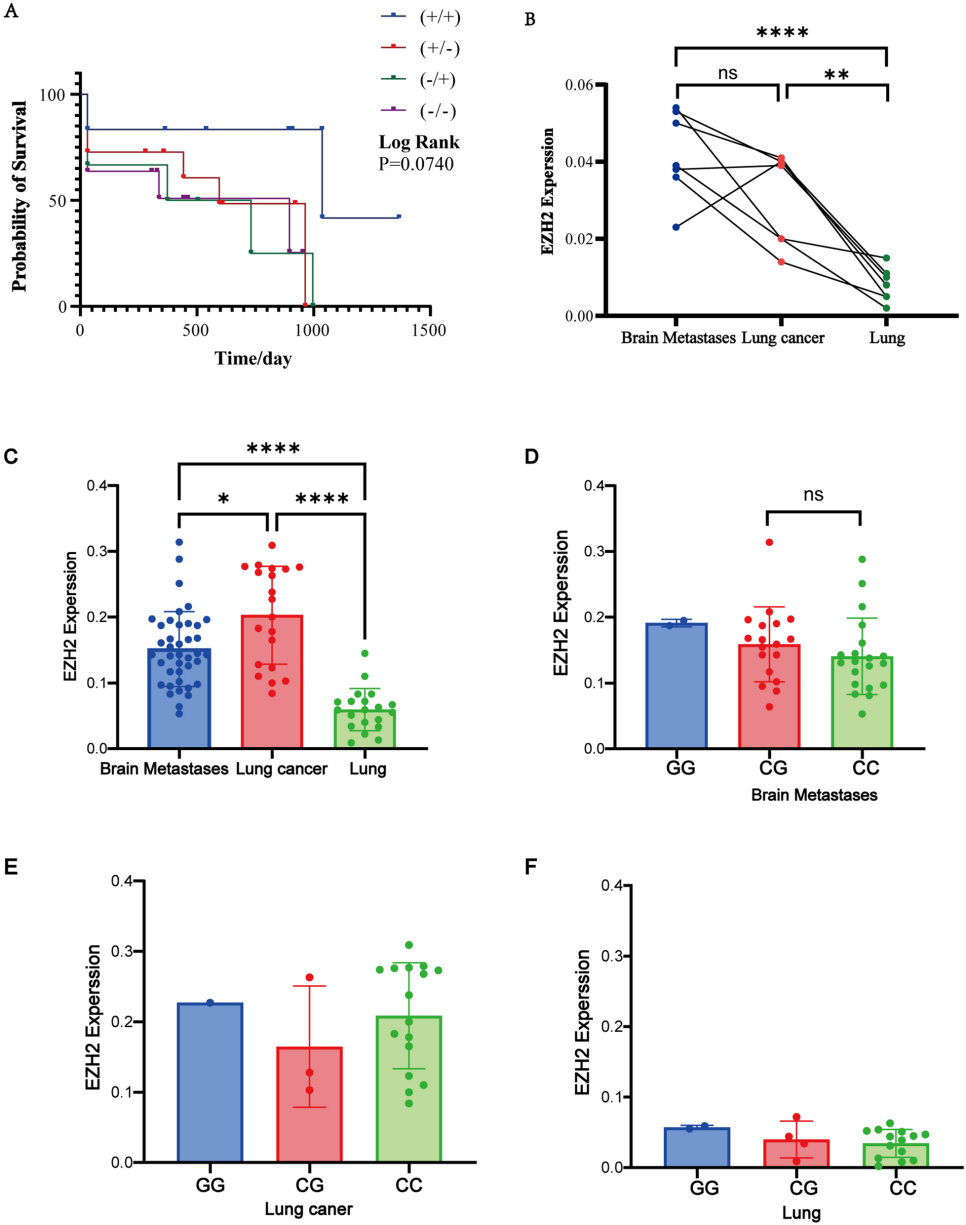
Relationship between *G553C* polymorphism of *EZH2* and *EZH2* expression and patient survival time. **A** Relationship between survival time of *EZH2* positive patients with different genotypes and histochemistry; **B** Expression of *EZH2* protein in 7 paired lung cancer brain metastases, lung cancer, and surrounding normal lung tissue samples. **C** Expression of *EZH2* protein in 40 lung cancer brain metastasis specimens and 20 lung cancer and adjacent lung tissue specimens. **D** The expression of three different genotypes of *EZH2* protein in the metastatic tissue of lung cancer patients with brain metastasis. **E** The expression of three different genotypes of *EZH2* protein in lung cancer tissues of lung cancer patients. **F** Three different genotypes of *EZH2* protein expression in lung cancer adjacent tissues of lung cancer patients (* *p* < 0.05, **p < 0.01, **p < 0.0001, ns no significant difference)

Among the selected cases of lung cancer brain metastasis in the study, 78 samples had complete follow-up data. Three different genotypes at the G553C locus were detected in the first-generation sequencing of lung cancer brain metastasis samples. Analysis showed that GG genotype and GC genotype were risk factors for lung cancer brain metastasis.According to *EZH2* mutation and immunohistochemical expression, the patients were divided into four groups. Using Kaplan Meier survival analysis,Log Rank (*p* = 0.0740) and Breslow (*p* = 0.3975) test showed that there was no significant difference among the genotype groups(Fig. 4A). According to the Welch ANOVA test analysis of *EZH2* expression in the three groups, the expression level of *EZH2* protein in lung cancer tissue was significantly increased compared to adjacent lung tissue (*p* = 0.0012), while there was no statistically significant difference between brain metastasis tissue and primary lung cancer tissue (*p* = 0.1061). The expression level of brain metastasis tissue samples from the same patient was significantly increased compared to adjacent lung tissue (*p* < 0.0001) (Fig. 4B).

Obtain the average optical density value of each specimen slice through Image Pro Plus software, which represents the expression level of *EZH2* protein. According to the Welch ANOVA test, the expression of *EZH2* in the brain tissues of 40 lung cancer patients with brain metastasis and 20 lung cancer patients with lung cancer and adjacent lung cancer tissues in three groups of samples will be analyzed. Compared with adjacent lung cancer tissues, the expression level of *EZH2* in lung cancer tissues is significantly increased (*p* < 0.0001), and the expression level is higher than that in brain metastasis tissues of lung cancer patients (*p* = 0.0309). Similarly, the expression level of *EZH2* in brain metastatic tissue samples of lung cancer patients with brain metastasis was significantly higher than that in adjacent tissue samples of lung cancer patients (*p* < 0.0001) (Fig. 4C).

Further analysis was conducted on the expression levels of *EZH2*, three different genotypes of GG, CG, and CC, in three groups of samples: lung cancer brain metastasis tissue, lung cancer tissue, and lung cancer adjacent tissue. Due to the limited number of GG genotypes, statistical analysis was not possible. In lung cancer brain metastasis tissue, T-tests were performed on the expression levels of *EZH2* between CG genotype and CC genotype. The results showed that there was no difference in expression levels between these two genotypes, while in other samples, due to the small number of GG and CG genotypes, statistical analysis was not conducted (Fig. 4D–F).

### RNA in situ hybridization expression

**Fig. 5:**
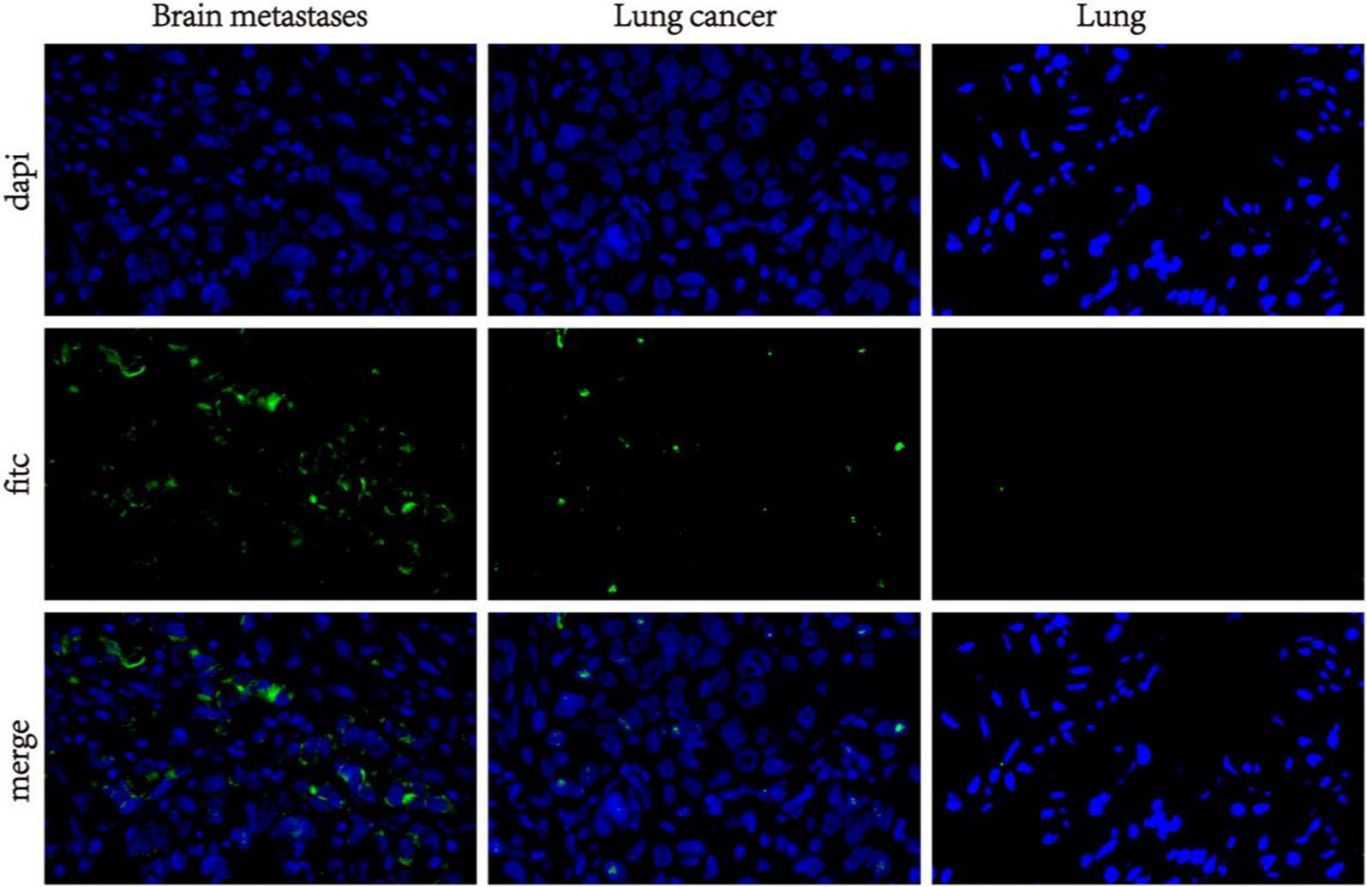
*EZH2* RNA in situ immunohybridization of lung cancer brain metastases, lung cancer, and adjacent tissues (× 400)

Six cases of brain metastases from lung cancer, six cases of lung cancer and adjacent lung cancer specimens were selected, including 2 cases of GG genotype, 2 cases of CG genotype and 2 cases of CC genotype. Two patients with brain metastases from CG type lung cancer were male, 67 years old, with adenocarcinoma and multiple brain metastases. A 61 year old male presented with solitary brain metastases from adenocarcinoma. Two patients with brain metastasis from GG type lung cancer were male, 57 years old, with adenocarcinoma and single brain metastasis.Male, 53 years old, adenocarcinoma, single brain metastasis. Two patients with brain metastasis from CC type lung cancer were male, 66 years old, with adenocarcinoma and single brain metastasis. Male, 60 years old, adenocarcinoma, multiple brain metastases. The specimens of 6 patients with lung cancer and adjacent cancer, 2 cases of CG type.Female, 59 years old, adenocarcinoma, lung cancer single focus.A 68 year old male presented with adenocarcinoma and lung cancer. Two cases of GG type were female, 68 years old, with adenocarcinoma and lung cancer. A 57 year old male presented with adenocarcinoma and lung cancer. Two cases of CC type were male, 63 years old, with multiple lesions of adenocarcinoma and lung cancer. A 44 year old male presented with adenocarcinoma and lung cancer.By observing the expression of *EZH2* RNA in situ hybridization under a fluorescence microscope, it was found that the lung tissue adjacent to lung cancer showed a low expression state, with a very small number of positive particles in the nucleus; the majority of cancer cells in lung cancer tissue express granular signal points in the nucleus; the expression of cancer cells in brain metastatic tissues of lung cancer is scattered and clustered with positive cytoplasm, with a higher degree of positivity; the positive expression pattern of *EZH2* reflects the progressive characteristics of *EZH2* positive expression pattern from adjacent lung tissue, lung cancer tissue to brain metastasis tissue. In the primary lesion before metastasis, there are scattered positive particles in the nucleus, and after metastasis, there are clustered positive signals in the cytoplasm (Fig. 5). By observing the positive signals of *EZH2* RNA in situ hybridization according to the expression depth and characteristics, the state of *EZH2* in the host can be determined. We found that as the disease progresses and metastasizes, the positive sites of *EZH2* RNA in situ hybridization move from the nucleus to the extracellular domain, and the degree of signal expression gradually increases.

## Discussion

Lung cancer is one of the main causes of death from malignant tumors, both in China and globally [11]. Brain metastasis can cause serious cognitive impairment, seriously affecting the survival and survival of patients. Therefore, the treatment of lung cancer patients largely fails due to tumor metastasis [12].

In this study, we sequenced brain metastasis tissue samples, lung cancer tissue samples, and lung cancer adjacent tissue samples from 5 patients with lung cancer brain metastasis through second-generation sequencing. We identified 14 differentially expressed genes and analyzed the function of each gene to identify the PCG protein family that plays a role in hematopoiesis and the nervous system, the gene *EZH2* involved in its encoding was found in 3 out of 5 patient samples, which resulted in new *EZH2* mutation sites after brain metastasis. To verify the results of second-generation sequencing, Sanger sequencing was performed on the metastatic tissue DNA of 5 lung cancer brain metastasis patients who were sent for second-generation sequencing. Ch7:148525904: C/G (GRCh37), also known as Ch7:148828812: C/G (GRCh38), was verified, which is also the SNP site rs2302427 (*EZH2*-G553C).

To explore the relationship between *EZH2*_G553C polymorphism and the occurrence, development, and prognosis of lung cancer brain metastasis in the Chinese population, 78 lung cancer brain metastasis specimens and 60 lung cancer specimens were tested for G553C locus. In the distribution of *EZH2*_G553C genotype, there is a significant difference between lung cancer brain metastasis and lung cancer population. The frequency of G allele frequency has a significant difference between lung cancer brain metastasis and lung cancer. The risk of lung cancer brain metastasis of G allele carriers has increased by 2.1241 times.This is the first study to discover the relationship between gene polymorphism in the exon region of the *EZH2* gene and the risk of brain metastasis in lung cancer. Our experimental results show that the gene frequency distribution in both the lung cancer brain metastasis group and the lung cancer group conforms to the Hardy Weinberg equilibrium, indicating that although our experiment is a hospital-based case control study, the selected samples are representative of the population. In addition, it also indicates good reproducibility of genotype and reliable sequencing validation results. Therefore, it can be concluded that the positive results obtained in this experiment are not caused by selective bias.

*EZH2* is an important component of the core protein complex 2 (PRC2) function. PCG proteins play a role in multiple protein complex families, including core protein complex 1 (PRC1) and core protein complex 2 (PRC2). In mammals, PRC2 is mainly composed of zeste enhancer (*EZH2* or EZH1), zeste 12 inhibitor (SUZ12), embryonic ectoderm development protein (EED) and retinoblastoma binding protein (RbAp46/48) [13]. *EZH2* is the core component of PRC2 catalytic activity, and *EZH2* (or related EZH1) is considered the only methyltransferase that mediates H3K27 methylation from low to high levels. It is known that the overexpression or mutation of *EZH2* is related to several invasive tumor types, such as prostate cancer, breast cancer and different types of lymphoma, and indicates that the prognosis of patients is poor [14–16]. Overexpression of *EZH2* can functionally affect (1) adhesion molecules, (2) cell cycle regulatory factors, (3) transcription factors, and (4) downregulation of DNA repair mechanisms [17–20], which have significant implications in cell transformation and metastasis.

In patients with brain metastasis of lung cancer, the genotype of G553C site of *EZH2* gene is GG. In one patient, the genotype of primary lung cancer puncture was GC, and there was a missense mutation in exon 15 of BRAF gene. After receiving pemetrexed + carboplatin + bevacizumab combined chemotherapy, the genotype was found to be GG genotype in brain metastasis after the progress of primary lung cancer GC. Another patient, with genotype CG in the biopsy specimen of the primary lung cancer lesion, underwent resection of the lung cancer brain metastasis specimen after 1 month without treatment. The metastatic lesion specimen was wild-type C. However, Gao et al. [21] found that the tumor survival rate of genotype G carriers at the G553C locus of the *EZH2* gene in liver cancer was higher than that of wild-type C carriers. It is clearly confirmed that the G553C point mutation of *EZH2* in hepatocellular carcinoma is a significant protective genetic change. In this study, patients receiving chemotherapy underwent a GC genotype mutation at the G553C locus of the *EZH2* gene in the primary lung cancer lesion after progression, while patients who did not receive treatment only showed a C genotype in brain metastasis resection samples one month later. There is reason to suspect that lung cancer patients who received chemotherapy developed a protective G mutation. Moreover, through Sanger sequencing detection, we found a sample with a frameshift mutation in the sequence following G553C after metastasis. This patient is a 64-year-old female with a primary lesion of multiple adenocarcinomas in the right lung, with a maximum diameter of 5.2cm. The metastatic lesion is a single adenocarcinoma in the cerebellum, with a maximum diameter of 5cm. The patient was lost after April 5, 2022. Compared to the primary site, the gene undergoes new changes after metastasis, which is consistent with the results of Jones team’s [22] analysis of matched primary and metastatic samples, demonstrating that metastasis typically achieves new changes through increased chromosomal stability and systemic treatment.

In our study, Kaplan Meier survival analysis of 78 patients showed no significant differences among genotype groups (Log Rank test *p* = 0.1054). However, patients with CC genotype have a higher number of deaths within 3 months compared to patients with GG + GC genotype, indicating that G553C of *EZH2* gene may affect patient prognosis, but it has not yet reached statistical significance. This may be due to differences in research results caused by insufficient sample size. Further research on the relationship between *EZH2* gene frequency and prognosis of lung cancer brain metastasis needs to be expanded in the future.

In the ENSEMBL database, the G allele frequency of the *EZH2* G553C locus in East Asia is the highest in the five regions with 0.206 among the data of the five regions in the world, while in the three regions of China, the G allele frequency of the Dai people in Xishuangbanna is as high as 0.269. The overall frequency shows that the G allele frequency of the G553C locus of the *EZH2* gene in Chinese normal people is at a high level in the world, Further research is needed to determine whether this is related to the occurrence and development of lung cancer and brain metastasis in the Chinese population.

Immunohistochemistry was observed in 40 cases of brain metastases from lung cancer and 20 cases of lung cancer and adjacent tissues. Compared with the adjacent lung tissues of lung cancer patients, the expression level of *EZH2* was significantly increased in lung cancer tissues, and the expression level was higher than that of brain metastases from lung cancer patients. Similarly, the expression level of *EZH2* in brain metastases from lung cancer patients was significantly higher than that of adjacent tissues from lung cancer patients. Carmen Behrens et al. [23] found that the immunohistochemical expression of *EZH2* in lung cancer brain metastasis was significantly higher than that in primary lung tissue. Similarly, in our 7 paired lung cancer brain metastasis tissues, lung cancer tissues, and lung cancer adjacent tissues, *EZH2* expression was found to be in the vicinity of the cancer, and the expression in the primary and post metastasis tissues showed a gradually increasing trend. Through statistical analysis of mean optical density values, it was found that, there is a significant difference in *EZH2* between adjacent lung cancer tissues and lung cancer tissues in the same patient.However, there was no statistically significant difference in expression between lung cancer tissue and lung cancer brain metastasis tissue, which may be due to a small number of paired samples. Yan et al. [24] found that *EZH2* immunohistochemical expression was higher in papillary thyroid carcinoma (PTC) than in surrounding normal tissues, and there were significant differences between *EZH2* and lymph node metastasis, extrathyroid involvement, and tumor multifocal growth in patients. Raahorst et al. [25] found that *EZH2* was not expressed in normal breast tissue by comparing the expression level of *EZH2* in normal breast tissue, invasive breast cancer and precancerous lesions, and *EZH2* was not expressed in ductal hyperplasia, well differentiated ductal carcinoma in situ and well differentiated invasive cancer, but only highly expressed in poorly differentiated breast cancer, These results indicate that the imbalance of *EZH2* expression is related to the loss of differentiation and development of human poorly differentiated breast cancer.Further use of RNA in situ immune hybridization technology to detect *EZH2* expression at the mRNA level revealed a progressive pattern of *EZH2* positive expression from surrounding lung tissue, lung cancer to brain metastasis. In the primary lesion before metastasis, there were scattered positive particles in the nucleus, while after metastasis, there were patchy positive signals in the cytoplasm. From lung cancer to the progression of brain metastasis, the positive sites of *EZH2* RNA in situ hybridization gradually increase in signal intensity from within the nucleus to outside the nucleus.

Based on the above analysis, the study found that the CG + GG genotype of the G553C locus of the *EZH2* gene had significant statistical differences between lung cancer brain metastasis and lung cancer tissue, and the difference between C and G allele frequency was statistically significant, and G allele carriers were more prone to brain metastasis. From the levels of protein and mRNA expression, it was found that the same patient showed an upward trend in expression from the adjacent lung cancer, primary lung cancer to metastatic lesions. Our conclusion explains for the first time the relationship between the *EZH2*_G553C polymorphism and the risk of lung cancer brain metastasis in the Chinese population, suggesting that the *EZH2*_G553C polymorphism may be associated with the occurrence and development of lung cancer brain metastasis.However, considering that the total number of brain metastasis samples selected is not large and only one locus is analyzed, it is not yet possible to label it as a complete label of the entire *EZH2* gene to explain the specific relationship between SNP polymorphism of the *EZH2* gene and the occurrence and development of lung cancer brain metastasis. We need to further increase the sample size, increase the number of loci, and conduct functional research on its locus polymorphism, thus providing more reliable evidence for the relationship between *EZH2* SNP polymorphism and the occurrence and development of brain metastasis in lung cancer. The Chinese society of Clinical Oncology (CSCO) guidelines for the diagnosis and treatment of lymphoma were updated. Tazemetostat, an *EZH2* inhibitor, was recommended in the guidelines for the first time. Tazemetostat is an inhibitor of methyltransferase *EZH2* and some *EZH2* function acquired mutations, including Y646X, A682G and A692V [26]^.^ Our study can be used as an index to prevent brain metastasis of lung cancer and predict the prognosis of patients, and provide a new direction for the treatment of brain metastasis of lung cancer.

## What’s new?

High-throughput sequencing found that *EZH2* mutation was closely related to brain metastasis of lung cancer.

*EZH2* G553C polymorphism contributes to the prediction of brain metastasis of lung cancer, in which G allele carriers are more prone to brain metastasis.

Provide experimental basis for early application of *EZH2* inhibitors in patients with susceptibility genes for lung cancer brain metastasis.

### Supplementary Information

**Additional file1: Fig. S1** FAM92B showed negative expression in brain metastases, primary lesions, and adjacent tissues, while EZH2 showed positive expression in the same tissues. H&E staining and EZH2, FAM92B immunohistochemical staining of paired brain metastases, primary lung cancer and lung tissues adjacent to lung cancer in patients with brain metastases from lung cancer, with scales of 100 um (100x), 300 um (400X). A. Brain metastases from lung cancer (H&E staining); EZH2 staining of brain metastases from lung cancer; FAM92B staining in brain metastases of lung cancer (× 100) (× 400). B. Primary lung cancer tissues (H&E staining); EZH2 staining of primary lung cancer tissues; FAM92B staining in primary lung cancer tissues (× 100) (× 400). C. Adjacent lung tissue (H&E staining); EZH2 staining of lung tissue adjacent to cancer; Fam92b staining (× 100) (× 400) was performed in adjacent lung tissues. (PNG 8020 KB)

## Data Availability

All data and materials generated during and/or analysed during the current study are available from the corresponding author on reasonable request.
