# Supplementary figures and images for: EZH2 G553C significantly increases the risk of brain metastasis from lung cancer due to salt bridge instability
Source: Cancer Cell Int. 2024 May 19;24:175. doi: 10.1186/s12935-024-03362-w (PMC11103815; doi:10.1186/s12935-024-03362-w)

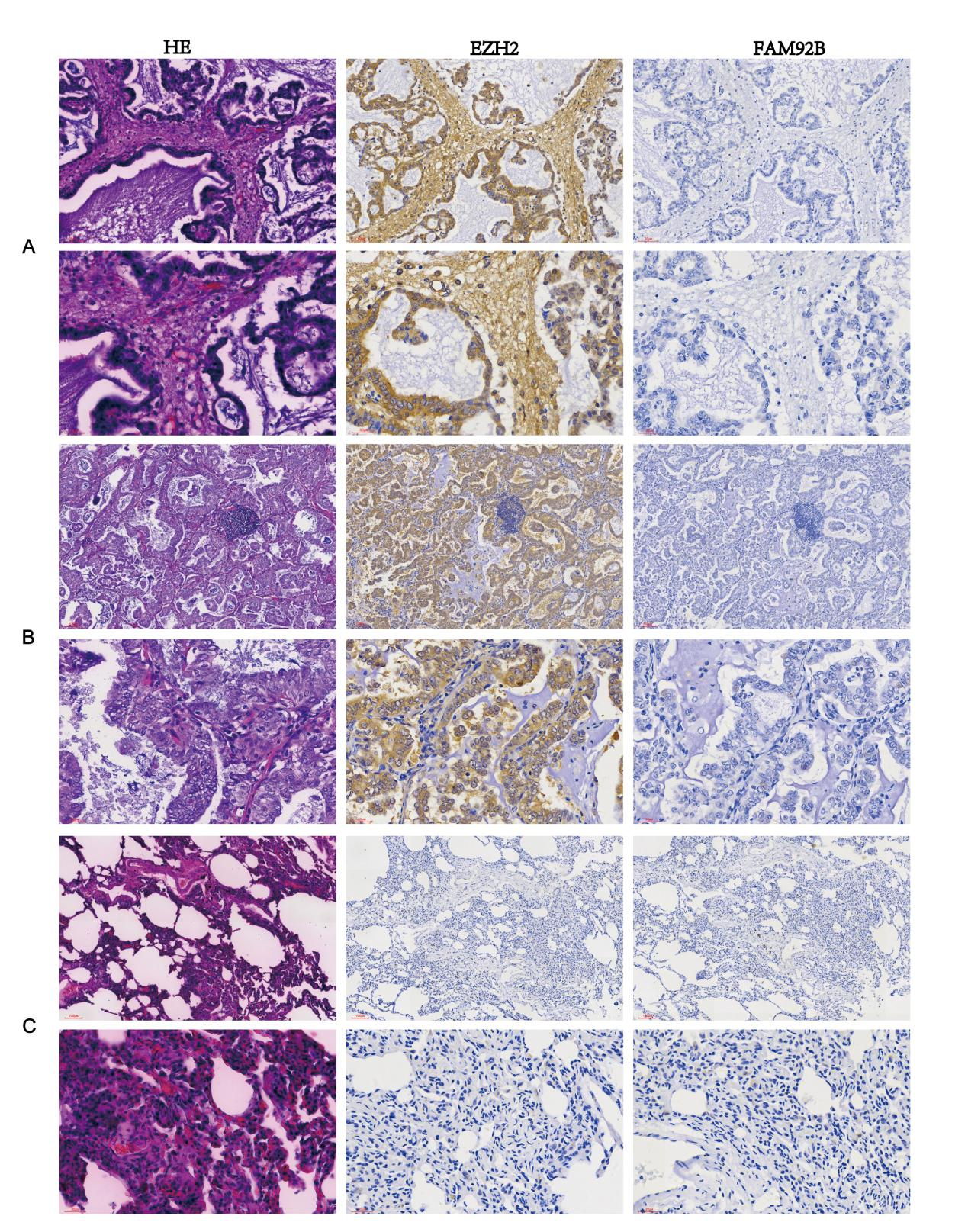

Supplement: Supplementary file 1 — Additional file1: Fig. S1 FAM92B showed negative expression in brain metastases, primary lesions, and adjacent tissues, while EZH2 showed positive expression in the same tissues. H&E staining and EZH2, FAM92B immunohistochemical staining of paired brain metastases, primary lung cancer and lung tissues adjacent to lung cancer in patients with brain metastases from lung cancer, with scales of 100 um (100x), 300 um (400X). A. Brain metastases from lung cancer (H&E staining); EZH2 staining of brain metastases from lung cancer; FAM92B staining in brain metastases of lung cancer (× 100) (× 400). B. Primary lung cancer tissues (H&E staining); EZH2 staining of primary lung cancer tissues; FAM92B staining in primary lung cancer tissues (× 100) (× 400). C. Adjacent lung tissue (H&E staining); EZH2 staining of lung tissue adjacent to cancer; Fam92b staining (× 100) (× 400) was performed in adjacent lung tissues. (PNG 8020 KB) [file 12935_2024_3362_MOESM1_ESM.png]
